# Comparisons of gut microbiota profiles in wild-type and gelatinase B/matrix metalloproteinase-9-deficient mice in acute DSS-induced colitis

**DOI:** 10.1038/s41522-018-0059-0

**Published:** 2018-09-03

**Authors:** Magali de Bruyn, João Sabino, Doris Vandeputte, Séverine Vermeire, Jeroen Raes, Ghislain Opdenakker

**Affiliations:** 1 Laboratory of Immunobiology, Department of Microbiology and Immunology, Rega Institute for Medical Research, KU Leuven, Herestraat 49, Box 1044, 3000 Leuven, Belgium; 2 Translational Research Center for Gastrointestinal Disorders (TARGID), Department of Clinical and Experimental Medicine, KU Leuven, Herestraat 49, Box 701, 3000 Leuven, Belgium; 3 Laboratory of Molecular Bacteriology, Department of Microbiology and Immunology, Rega Institute for Medical Research, VIB Center for Microbiology, KU Leuven, Herestraat 49, Box 1028, 3000 Leuven, Belgium; 4 Department of Gastroenterology and Hepatology, University Hospitals Leuven, Herestraat 49, 3000 Leuven, Belgium

## Abstract

Gut microbiota help to educate the immune system and a number of involved immune cells were recently characterized. However, specific molecular determinants in these processes are not known, and, reciprocally, little information exists about single host determinants that alter the microbiota. Gelatinase B/matrix metalloproteinase-9 (MMP-9), an innate immune regulator and effector, has been suggested as such a host determinant. In this study, acute colitis was induced in co-housed MMP-9^-/-^ mice (*n* = 10) and their wild-type (WT) littermates (*n* = 10) via oral administration of 3% dextran sodium sulfate (DSS) for 7 days followed by 2 days of regular drinking water. Control mice (10 WT and 10 MMP-9^-/-^) received normal drinking water. Fecal samples were collected at time of sacrifice and immediately frozen at −80 °C. Microbiota analysis was performed using 16S rRNA amplicon sequencing on Illumina MiSeq and taxonomic annotation was performed using the Ribosomal Database Project as reference. Statistical analysis correcting for multiple testing was done using R. No significant differences in clinical or histopathological parameters were found between both genotypes with DSS-induced colitis. Observed microbial richness at genus level and microbiota composition were not significantly influenced by host genotype. In contrast, weight loss, disease activity index, cage, and phenotype did significantly influence the intestinal microbiota composition. After multivariate analysis, cage and phenotype were identified as the sole drivers of microbiota composition variability. In conclusion, changes in fecal microbiota composition were not significantly altered in MMP-9-deficient mice compared to wild-type littermates, but instead were mainly driven by DSS-induced colonic inflammation.

## Colitis: a protein cleared of involvement

A protein that regulates aspects of the immune system has been proposed to influence gut microbial populations implicated in the inflammatory conditions known as colitis, but new evidence suggests the protein has no such effect. Ghislain Opdenakker and colleagues at the Rega Institute for Medical Research in Belgium examined the issue in mice with chemically induced colitis. The gut microbes of normal “wild-type” animals were compared with those in animals lacking the gene for the protein, “gelatinase B/matrix metalloproteinase-9”. The absence of the gene, and therefore of the protein it codes for, caused no significant alteration in the gut microbial population. The presence of colitis, however, did alter the gut microbial population relative to mice with no colitis. The results will assist work to understand the networks of cause and effect linking gut microbes and colitis.

## Introduction

Chronic immune-mediated conditions, including inflammatory bowel disease (IBD), are characterized by an interplay between genetic, immunological, and environmental factors.^1^ It is increasingly recognized that host gut microbiota constitute important factors in these diseases.^2–4^ Microbiota help to educate the immune system and codetermine autoimmune reactions. Whereas in these reactions the cellular elements are well studied,^5^ specific molecular targets are poorly defined. This implies that, whereas cellular targeting is well defined for the development of novel therapies, molecular targeting needs much more research. With the use of mouse models of colitis it has been shown that heterogeneity in age, gender, genetic background, and type of colitis models may give contradicting observations and difficulties to extrapolate findings to the human situation.^6–9^ Nevertheless, animal models may provide valuable information as the experimental conditions can be well controlled.

Supplementation of dextran sodium sulfate (DSS) to the drinking water of mice is commonly used to induce experimental colitis as a model for IBD to investigate host factors contributing to IBD and potential new therapeutic targets. Importantly, oral DSS supplementation promotes gut microbial dysbiosis in mice with a decrease in microbial richness.^10–13^ Microbial and immunological changes seem to appear before the development of colitis, indicating that these changes may play a role in the potentiation of the abnormal inflammatory response seen in DSS-treated animals.^12^

A possible role of MMP-9 in bacterial-dependent or bacterial-induced models of intestinal inflammation has been described in several studies.^14–19^ Heimesaat et al. investigated the effect of MMP-2 and MMP-9 deficiency on the development of acute DSS-induced colitis and concluded that MMP-2 plays a causal role in the establishment of colitis.^14^ In addition to other lowered inflammatory parameters, the authors observed less overgrowth of the colon by *Escherichia coli* (*E. coli*) in MMP-2 knockout mice, but not in MMP-9 knockout mice. Moreover, blocking gelatinases (MMP-2 and MMP-9) with a selective gelatinase inhibitor (RO28-2653) also resulted in less overgrowth of *E. coli*.^15^ The effect of gelatinase deficiency or blockage was further studied in models of bacterial-induced intestinal inflammation.^16–18^ Consistent with previous studies, MMP-2 seemed to play a more prominent role than MMP-9 in mediating bacterial-induced intestinal immunopathology. Finally, an infectious model with *Citrobacter rodentium* (*C. rodentium*) was used to induce colitis in MMP-9 knockout mice.^19^ Consistent with our recent data on MMP-9 knockout mice in DSS-induced and TNBS-induced models of inflammatory colitis,^9^ similar development of inflammatory parameters was observed in MMP-9 knockout and WT mice. Furthermore, the authors showed that MMP-9 knockout mice had lower colonization levels of *C. rodentium*, were protected from reductions in fecal microbial alpha diversity, and had a larger population of segmented filamentous bacteria. It is well known that MMP-9 alters immune functions^20^ and that altered microbiota affect the immune system.^5^ Therefore, it was important to study whether host factors, such as MMP-9, induce alterations in microbiota. However, in all the mentioned studies, sequencing techniques for a complete assessment of microbiota composition were lacking. In the present study, we used 16S rRNA gene sequencing to investigate the effect of MMP-9 deficiency on fecal microbiota in control conditions and after induction of acute colitis with DSS.

## Results

### Development of colitis in MMP-9^-/-^ mice and WT littermates

**Fig. 1:**
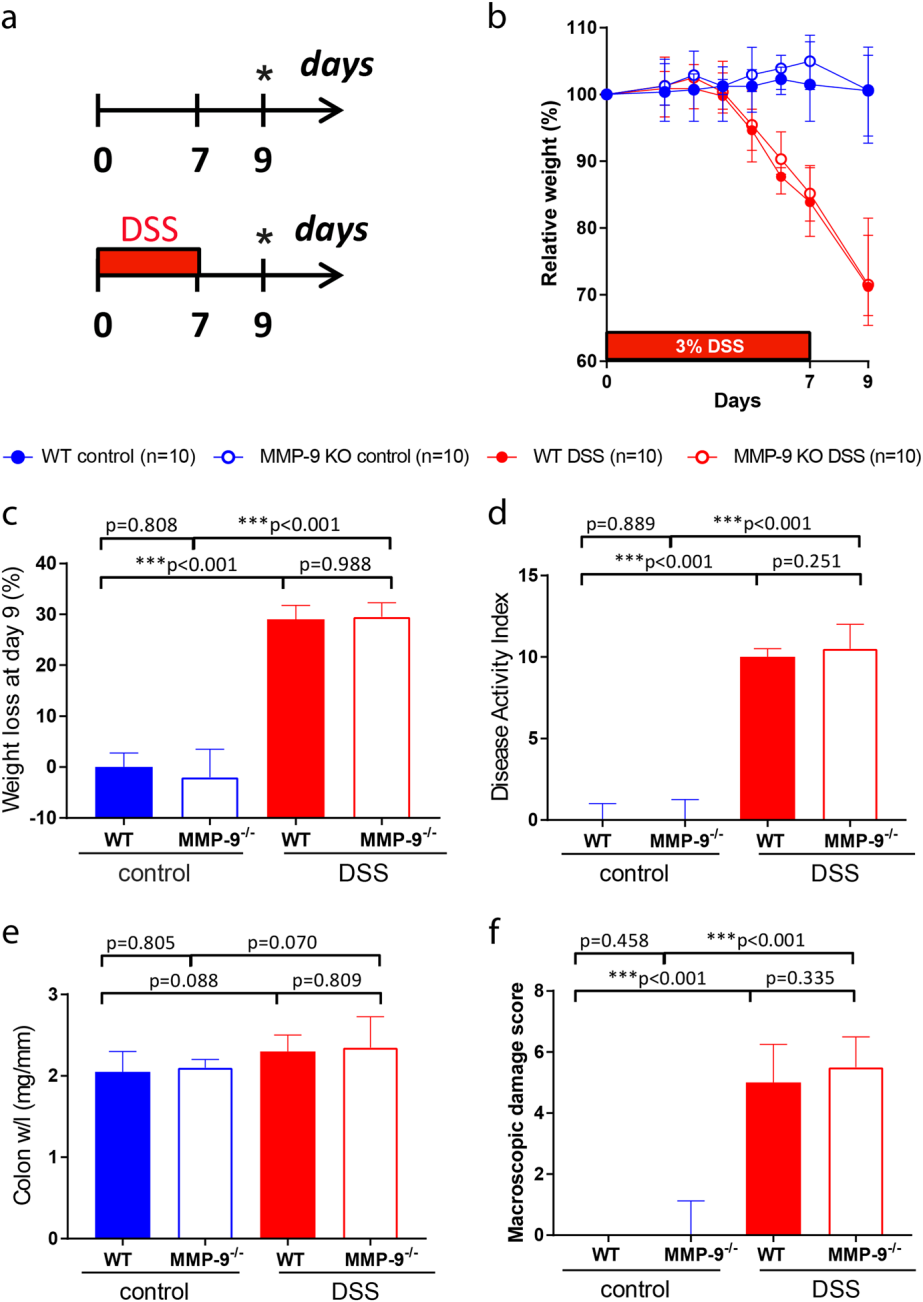
Clinical and macroscopic parameters of DSS-induced inflammation in WT and MMP-9^-/-^ mice. **a** Representation of the acute DSS model. DSS-exposed mice comprised of WT mice (*n* = 10) and MMP-9^-/-^ mice (*n* = 10). Control mice received normal drinking water throughout the experiment (WT mice (*n* = 10), MMP-9^-/-^ mice (*n* = 10)). All groups consisted of five males and five females. **b** Relative weight loss from the start of the experiment until the time of sacrifice (* day 9). **c** Absolute weight loss at the time of sacrifice. **d** Disease activity index including stools, blood, and weight loss at the time of sacrifice. **e** Colon weight/length (w/l) ratio. **f** Macroscopic damage score including mesenterial colonic adhesion, hyperemia along the colon, and length of colonic inflammation. Medians with interquartile ranges are shown and Mann–Whitney U tests were performed (***p < 0.001)

In contrast to published data,^21–23^ we did not observe protective effects by MMP-9 gene deletion on colitis phenotypic scores. Both MMP-9^-/-^ mice and WT littermates developed severe acute colitis after DSS administration with loss of body weight (Fig. 1a, b, c), higher disease activity index (DAI) (Fig. 1d), increased colon/weight length ratio (Fig. 1e), and elevated macroscopic damage score (Fig. 1f) compared to corresponding control mice.

### General characteristics of the microbiota in MMP-9^-/-^ mice and WT littermates

**Fig. 2:**
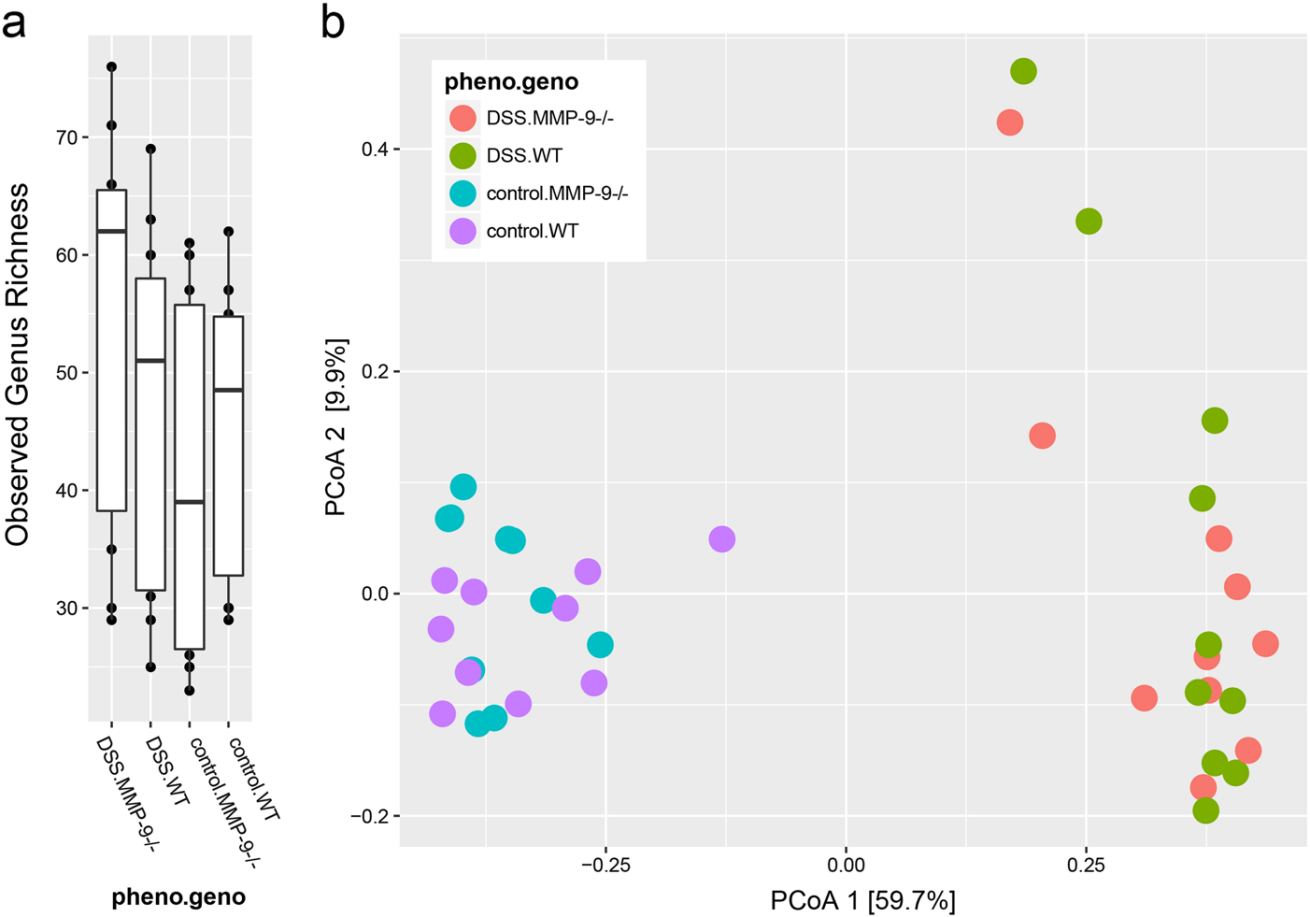
Fecal microbiota diversity in control and DSS-treated MMP-9^-/-^ mice and WT littermates. **a** Box plot representation of microbiota richness (number of observed operational taxonomic units [OTUs] per sample) distribution across MMP-9^-/-^ and WT mice. **b** Variation in microbial community composition represented by a principal coordinates analysis (PCoA) of the Bray–Curtis dissimilarity matrix, calculated from the OTU-level relative abundance matrix. Pheno.geno indicates the combination of phenotype (control or DSS) and genotype (WT or MMP-9^-/-^) within each of the four experimental groups

The observed microbial richness (alpha diversity) at genus level was not significantly different between both genotypes (Mann–Whitney U test, *p* = 0.756). Moreover, no differences were found in observed microbial richness at genus level between both phenotypes (Mann–Whitney U test, *p* = 0.088) (Fig. 2a). As shown in the principal coordinates analysis (PCoA) plot (Fig. 2b), the overall composition of the microbiota in MMP-9^-/-^ and WT mice was mainly driven by phenotype (control vs. DSS) (Adonis test, *p* = 0.001), whereas genotype (WT vs. MMP-9^-/-^) had no influence (Adonis test, *p* = 0.996). The variability in microbiota composition was smaller in the control group compared to the DSS group.

### Contribution of different bacterial genera to microbiota composition

**Fig. 3:**
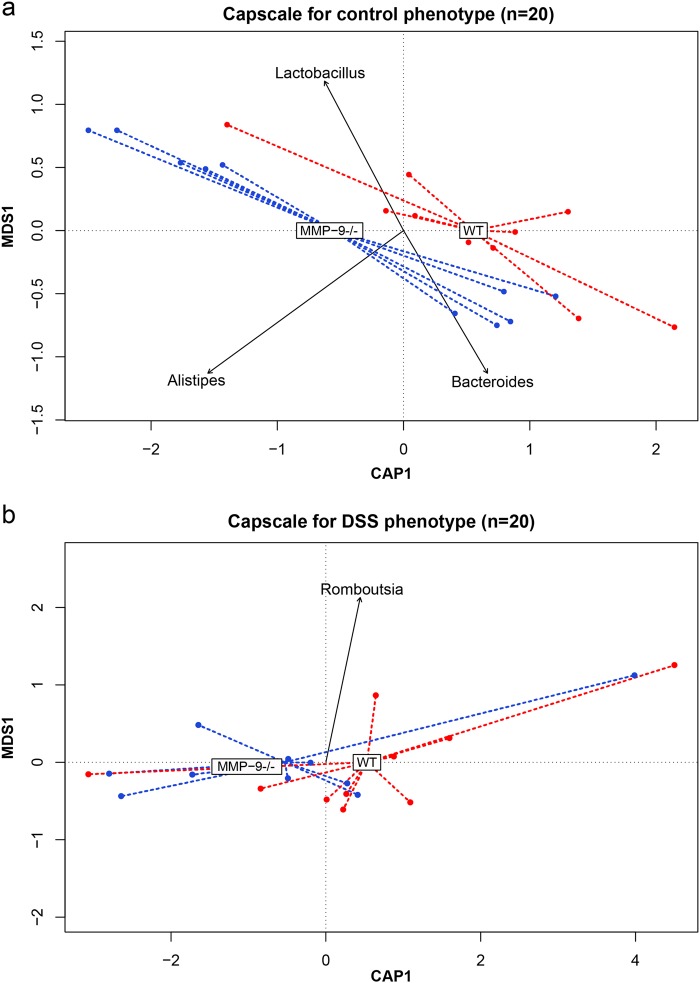
Analysis of the microbiota composition with genotype as constrained variable. Differences in microbiota composition between WT and MMP-9^**-/-**^ mice within the control **a** and DSS **b** phenotype. The plots show the distance-based redundancy analysis performed with capscale function (vegan package). Dots represent individual samples plotted according to their specific CAP1 and MDS1 coordinates and the dotted lines connect the samples with their corresponding genotype (MMP-9^-/-^ in blue and WT in red). Genera with a |CAP| > 0.1 are indicated on the plot and the arrow represents the CAP1 and MDS1 coordinates of each genus. CAP canonical analysis of principal coordinates, MDS multidimensional scaling

The main genera contributing to the variability in the overall microbiota composition were *Bacteroides*, *Clostridium XI*, *Clostridium sensu stricto*, *Enterococcus*, *Enterorhabdus*, *Romboutsia*, and *Turicibacter* (PCoA, envfit *r*^2^ > 0.5, *p* = 0.001). Analysis of the overall microbiota composition, using genotype as constrained variable, identified the main genera contributing to the separation of the samples within the control (WT control vs. MMP-9^-/-^ control) or DSS group (WT DSS vs. MMP-9^-/-^ DSS) (capscale). The genera *Bacteroides*, *Alistipes*, and *Lactobacillus* explained most of the variability of the microbiota composition between WT and MMP-9^-/-^ in the control group (Fig. 3a). In the DSS group, the genus *Romboutsia* explained most of the variability between WT and MMP-9^-/-^ mice (Fig. 3b).

### The effect of DSS on microbiota in MMP-9^-/-^ mice and WT littermates

**Fig. 4:**
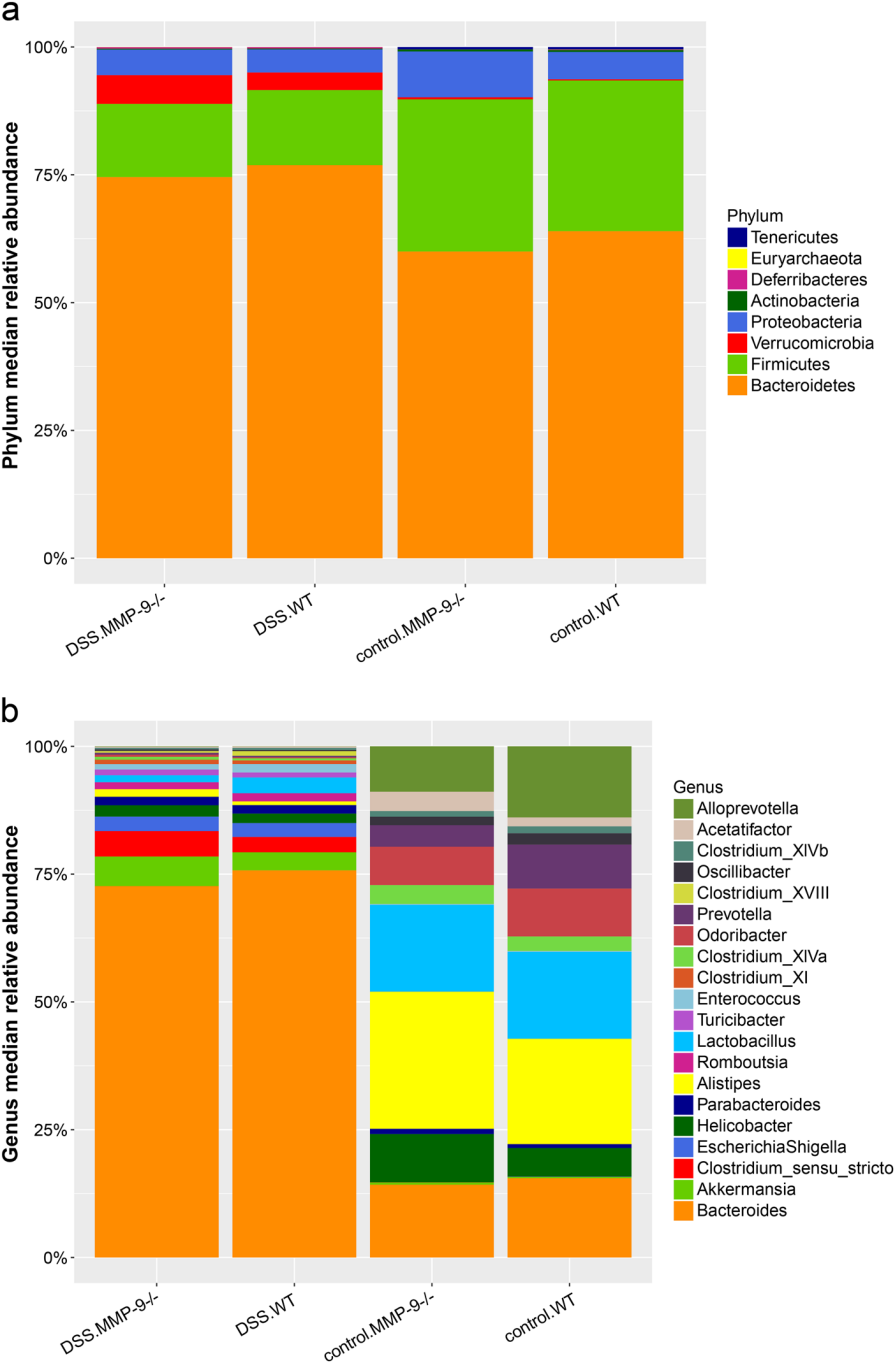
Fecal microbiota profiles in control and DSS-treated MMP-9^-/-^ mice and WT littermates. Relative abundances at phylum level **a** and at genus level **b** are shown for the four experimental groups (DSS.MMP-9^-/-^, DSS.WT, control.MMP-9^-/-^, and control.WT). The top 20 most abundant genera are represented

**Fig. 5:**
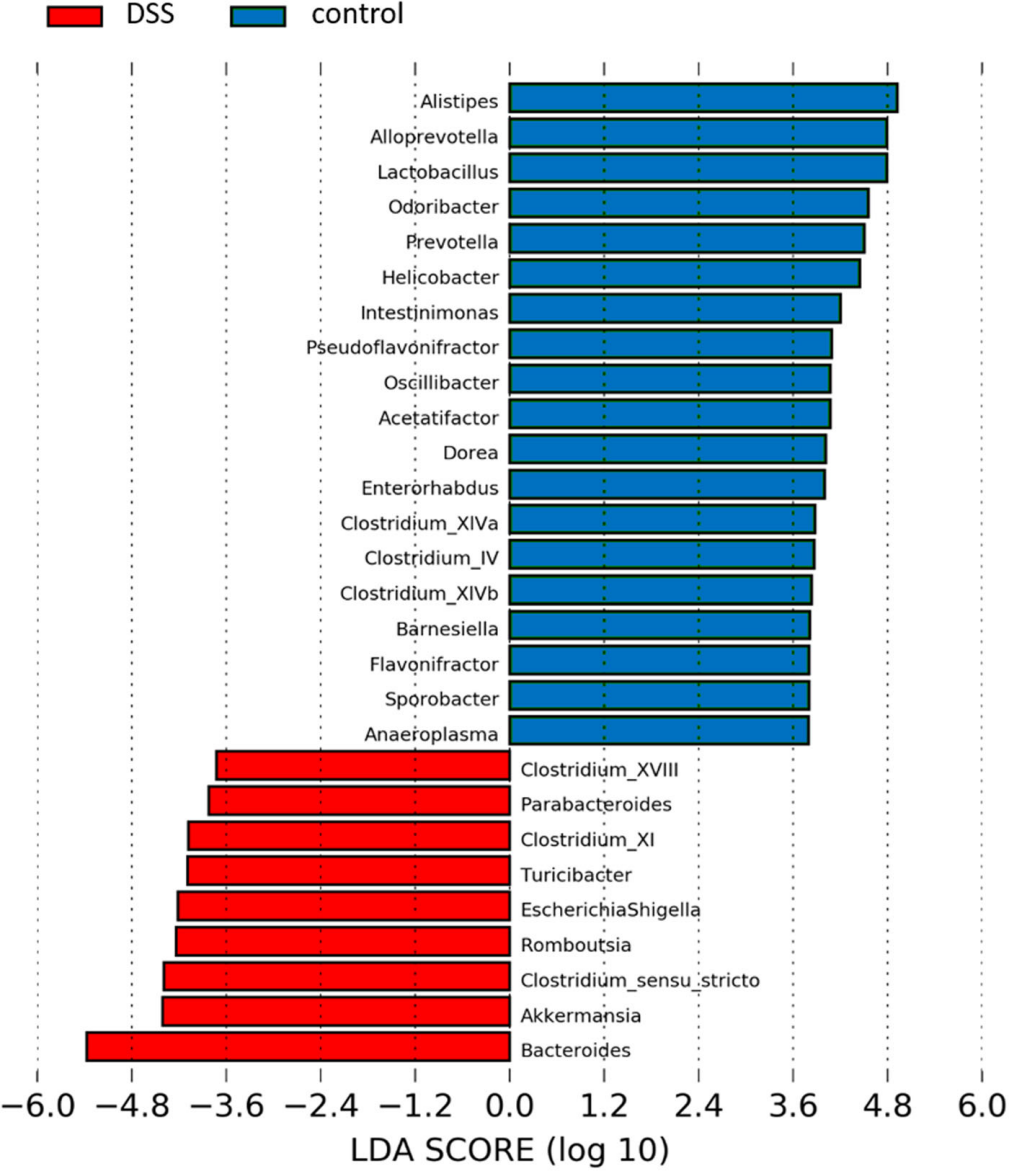
Differences in microbiota composition at genus level between control and DSS-treated mice with LEfSe (Linear discriminant analysis Effect Size). Genera indicated in blue were more abundantly present in control mice, whereas genera in red were found to be more abundant in mice with DSS-induced colitis. No differences were observed when subdividing according to genotype (WT or MMP-9^-/-^). LDA linear discriminant analysis

Both at phylum (Fig. 4a) and at genus level (Fig. 4b), the composition of the microbiota was strongly influenced by DSS. At phylum level, the most abundant phyla in WT vs. MMP-9^-/-^ mice included Bacteroidetes, Firmicutes, Proteobacteria, and Verruccomicrobia. Mice that received DSS showed higher relative abundances of Bacteroidetes and Verrucomicrobia and lower relative abundances of Firmicutes and Proteobacteria in comparison with corresponding control mice. At genus level, *Bacteroides* accounted for more than 70% of the identified bacterial genera in the DSS group (Fig. 4b). Besides *Bacteroides*, 29 other genera were significantly different between DSS and control mice (Mann–Whitney U test, false discovery rate (FDR) adjusted *p* < 0.05). These results were confirmed with Linear discriminant analysis Effect Size (LEfSe) (Fig. 5). However, no significant differences were found at genus level between WT and MMP-9^-/-^ mice.

### Covariates influencing the intestinal microbiota

**Fig. 6:**
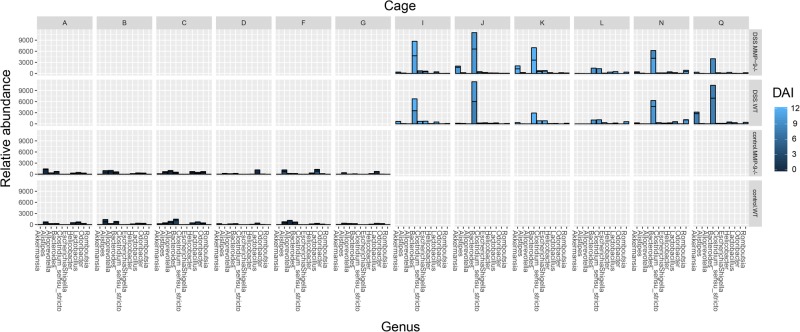
Drivers of changes in fecal microbiota composition at the genus level. Bar plot depicting the relative abundance of the 10 most abundant genera in all samples. Samples are divided per cage (A, B, C, D, F, G, I, J, K, L, N, and Q) and the four respective phenotype–genotype combinations (DSS.MMP-9^-/-^, DSS.WT, control.MMP-9^-/-^, and control.WT). The color intensities of the bars represent the disease activity index (DAI) with brighter colors indicating higher inflammatory burden. Each bar of the histogram represents the cumulative relative abundance of all samples within one group and the line within each bar indicates the separation of each sample within the group. If no line is observed within the bar, only one sample was available

In order to study the influence of covariates, we tested the correlation of all 14 metadata variables with overall microbiota composition. Absolute weight loss, DAI, phenotype, and cage were identified to correlate with the microbiota composition (PCoA, envfit, *r*^2^ > 0.8, *p* < 0.001). These covariates were then included in a forward stepwise redundancy analysis on genus-level PCoA (Bray–Curtis dissimilarity) in order to identify non-redundant covariates. Cage had a non-redundant effect size on microbiota composition of 63.86%. Gender did not significantly correlate with microbiota composition (PCoA, envfit, *r*^2^ = 0.0024, *p* = 0.877). In addition, association between metadata variables and microbial community relative abundance at genus level was performed with multivariate statistical framework (MaAsLin), isolating the influence of possible confounders. Cage and phenotype were the only variables that remained significantly associated with microbial genera (Fig. 6).

## Discussion

It is increasingly recognized that many knockout studies in mice cannot be reproduced, not only due to the failure of the knockout by homologous recombination, but because of the lack of stringent control conditions.^7,8^ In some cases, as described for MMP-9,^24^ it turns out that the knockout is leaky and thus still produces the intact protein of interest. Moreover, the genetic background of the wild-type mice is often not well controlled, thus providing phenotypic differences which are not caused by the knockout, but because of other gene differences. Importantly, environmental factors, of which microbiota are shown to be important, can also influence phenotypes. Gene knockout studies may therefore be confounded when the knockout and wild-type mice come from different (commercial) sources, with different dietary food and fluid intake and different microbiota. We recently demonstrated that induction of colitis with DSS was not attenuated in MMP-9 knockout mice compared to WT mice.^9^ These data are in contrast with current literature and we believe this is due to several factors, as described previously.^9^ In addition to optimal control for genetic background and leakiness of the used mice, the aim of the current study was to investigate the influence of the environment on our results and, vice versa, the influence of MMP-9 gene deficiency on the composition of the microbiota.

As described earlier, we did not observe any differences in infections or seroconversion against a number of classically tested animal pathogens between sentinel WT and MMP-9^-/-^ mice from the same unique breeding insulator over a period of 15 years.^9^ During our colitis experiments, WT and MMP-9^-/-^ mice were co-housed according to phenotype. This implicates that WT and MMP-9^-/-^ mice received the same drinking bottle with normal drinking water or water supplemented with DSS, which excludes potential DSS intake-related differences within the same cage. However, we did observe a significant effect of cage on the composition of the microbiota, more specifically with *Clostridium sensu stricto*. As shown in Fig. 6, the variation of microbiota composition was different between control and DSS cages, but also between cages that received DSS. This cage effect has already been described by Hildebrand et al., whereby caging contributed for 31.7% to the variance in microbiota composition in healthy mice.^25^ In our study, this was 63.86% for control and DSS-treated mice combined. This difference was probably due to the phenotype distribution according to cage. The effect of cage within the DSS phenotype was 44.9%. However, this did not significantly influence microbiota composition. The choice of co-housing does have its limitations, since a potential bias includes synchronization of microbiota between both genotypes due to coprophagia.^25^ This effect was previously described by several groups who demonstrated that microbiota-related phenotypes could be transferred between co-housed mice after several weeks of sharing a cage.^26–28^ This implies that if any subtle differences in microbiota composition would exist between both genotypes, we would possibly miss these due to fact that mice within the same cage will transfer their microbiota. However, since we showed that there is a large effect between cages (63.86%), we decided that co-housing of both genotypes was the correct experimental design for our study. This decision was also based on previous guidelines for experimental design that take into account the key variables that affect gut microbiota composition with respect to housing conditions.^7^ Additional studies are warranted to further evaluate the effects of co-housing and coprophagia on microbiota composition in mice with different genotypes.

Several microbial taxa contributed to the observed dysbiosis caused by DSS. At phylum level, we observed that DSS-supplemented mice had higher relative abundances of Bacteroidetes and Verrucomicrobia and lower relative abundances of Firmicutes and Proteobacteria in comparison with corresponding control mice. In a study by Nagalingam et al., higher relative abundancies of Verrucomicrobia and lower relative abundancies of Proteobacteria were also observed in mice with DSS-induced colitis.^12^ However, in contrast to our study, these authors observed decreased levels of Bacteroidetes and increased levels of Firmicutes. Moreover, they identified Firmicutes as the most abundant phylum, whereas this was the case for Bacteroidetes in our study. A potential explanation for the observed differences is the fact that Nagalingam and colleagues studied cecal tissue with T-RFLP analysis. In addition, our observations were in contrast to earlier published data by Munyaka et al., whereby a reduction in Bacteroidetes and an increase in Proteobacteria in colonic and fecal samples from acute DSS colitis were observed.^10^ However, many other aspects of this study were complementary to our data. For instance, these researchers also identified Firmicutes, Bacteroidetes, Proteobacteria, Deferribacteres, Tenericutes, and Verrucomicrobia as the six most abundant phyla, whereas Actinobacteria was identified as a low-abundant phylum. At genus level, *Bacteroides* was identified as the predominant genus involved in dysbiosis caused by DSS as it accounted for more than 70% of the identified bacterial genera. Furthermore, we observed that DSS-supplemented mice had lower relative abundances of *Alistipes*, *Alloprevotella*, *Prevotella*, *Odoribacter*, *Lactobacillus*, and *Helicobactor*; and higher relative abundances of *Romboutsia*, *Akkermansia*, *Escherichia*, *Shigella*, and *Clostridium sensu stricto* compared to control mice. The genus *Romboutsia* is a newly described bacterial genus, first described in 2014,^29^ which may explain the lack of evidence about this genus in the literature. Moreover, the family Enterobacteriaceae was significantly increased in mice that received DSS (*p* < 0.001) and a linear relationship was found between DAI and the incidence of Enterobacteriaceae (Spearman’s rho = 0.82, *p* < 0.001) (data not shown). These findings confirm previous observations described by Hakansson and colleagues.^11^

## Conclusions

In this study, we investigated changes in microbiota in co-housed WT and MMP-9^-/-^ mice in control and acute DSS-induced colitis conditions. Although limited to co-housed animals, we observed no effect of MMP-9 gene knockout on microbial profiles, whereas DSS had a considerable effect and caused dysbiosis in both WT and MMP-9^-/-^ mice. These data complement our earlier findings that MMP-9 gene knockout has no effect on development of colitis, and, in addition, has no influence on the composition of the intestinal microbiota.

## Methods

### Animals

MMP-9^-/-^ mice and their WT (C57BL/6J) littermates were selectively backcrossed by siblings over 13 generations^9^ and bred under specific pathogen-free conditions. All mice were transferred to conventional housing at 7 weeks of age and were co-housed 1 week prior to start of the experiment. Mice were raised under identical environmental conditions (e.g., 12 h light/dark cycle, identical food, and drinking water). All mice were confirmed for their MMP-9^-/-^ or WT genotype by PCR^30^ and the genetic background of the mice was carefully characterized by SNP analysis.^9^

The study was approved by the local ethics committee for animal experimentation of the University of Leuven (P178-2011) and was conducted following all relevant guidelines and procedures. Exclusion criteria included >30% weight loss and/or severe malaise of the animals.

### DSS-induced acute colitis

Acute colitis was induced as described previously.^31^ Briefly, 8–10-week-old male and female WT (*n* = 10) and MMP-9^-/-^ (*n* = 10) mice received 3% DSS (35–50 kDa; MP Biomedicals, Illkirch, France) in the drinking water during 7 days followed by 2 days on regular drinking water (Fig. 1a). Control WT (*n* = 10) and MMP-9^-/-^ (*n* = 10) mice received regular drinking water throughout the duration of the experiment. Each group consisted of five male and five female mice and sample size was chosen based on previous experimental experiences. Of importance, WT and MMP-9^-/-^ mice were co-housed (maximum *n* = 5 per cage) during the experiment ensuring identical environmental conditions as well as controlling for cage-related effects (e.g., coprophagia and intake of food and DSS-supplemented drinking water).

### Evaluation of body weight and colonic inflammation

Mice were weighed daily or every other day until time of sacrifice (day 9). Relative weight loss was calculated based on the weight of the mice at the start of the experiment and absolute weight loss was measured at time of sacrifice. The DAI was calculated based on stool consistency, presence of blood, and weight loss at time of sacrifice. As measurements of colonic inflammation, colon weight/length ratio, and macroscopic damage score (hyperemia along the colon, mesenterial colonic adhesion, and length of colonic inflammation) were included. The investigators were blinded for the genotype (WT or MMP-9^-/-^) of the mice.

### Fecal DNA extraction

Fecal samples were collected from the resected colon and stored at −80 °C within 2 h after sampling. The total amount of fecal content with a median (interquartile) weight of 88 (10–137.5) mg was used for bacterial DNA extraction. The PowerMicrobiome RNA Isolation Kit (Mo Bio Laboratories, Carlsbad, CA, USA) was used with an adapted protocol. Briefly, samples were homogenized with the use of mechanical beating in a lysis buffer containing 1% β-mercaptoethanol. In addition, the samples were incubated at 90 °C for 10 min. The supernatants containing DNA and RNA from both bacterial and murine cells were then bound to a spin filter membrane with the use of centrifugation. After three washing steps, the nucleic acids were eluted from the membrane in 100 µl RNase-free water and were stored at −80 °C. Bacterial DNA was quantified with a Qubit™ 2.0 fluorometer (Life Technologies, Grand Island, New York). After PCR amplification, Fragment analyzer™ (Advanced Analytical Technologies, Ames, Iowa) was used for quality control and quantification of the libraries.

### 16S rRNA gene-based microbiota profiling

The V4 region of the 16S rRNA gene was amplified with primer pairs 515F and 806R, with single multiplex identifier and adapters as described previously.^32^ Sequencing was performed on an Illumina MiSeq sequencer (MiSeq v2 kit, Illumina, San Diego, CA, USA) yielding 250 bp pair-ended reads. After de-multiplexing, fastq sequences were merged using FLASH software™ (V.1.2.10, Johns Hopkins University, Baltimore, USA)^33^ with default parameters. Combined reads quality threshold was set at minimum 30 quality score over 90% of read length (Fastx tool kit; http://hannonlab.cshl.edu/fastx_toolkit/) and chimeric sequences were filtered out (UCHIME^34^). Downsizing to 10,000 reads by random selection of quality-checked reads was performed for each sample. Ribosomal Database Project reference database (v2.12)^35^ was used for taxonomic assignment. The operational taxonomic unit (OTU)-level relative abundance matrix was obtained by de novo clustering of reads at 97% identity (USEARCH^36^).

### Statistical analyses

R software (v3.3.3) was used and non-parametric tests were performed unless specifically stated elsewhere. Shapiro–Wilk test was used to test continuous variables for normality. FDR correction was applied when needed. Adjusted *p*-values < 0.05 were considered significant. Student’s *t*-test was used to test the differences in microbiota species richness (metric = observed). PCoA with Bray–Curtis dissimilarity on genus-level community composition was used to visualize microbiota variation across samples (phyloseq package,^37^ version 1.16.2). Within the vegan package (version 2.4-2),^38^ vector fitting was performed with envfit function, community differences between groups were tested with Adonis non-parametric tests, and the contribution of different bacterial genera in the genus-level community composition was evaluated with correspondence analysis (rda function) and distance-based redundancy analysis (capscale function). Further analysis of the influence of genotype and phenotype in the intestinal microbiota was inferred with unconstrained and constrained partial least squares discriminant analysis (plsDA function, DiscriMiner package version 0.1-29). Correlation between taxa relative abundances and continuous metadata was performed with Spearman correlation. Differences in the taxa relative abundances between different groups were tested with Mann–Whitney U tests. These results were compared with LEfSe performed in the Galaxy platform, with the implementation of phenotype as class and genotype as subclass.^39^ Multivariate analysis was performed with MaAsLin R package V.0.0.3.^40^

### Data availability

Raw sequencing reads were deposited in the European Nucleotide Archive (PRJEB21337, http://www.ebi.ac.uk/ena/data/view/PRJEB21337). The metadata supporting the conclusions of this article are available MixS format as Supplementary File 1.

## Electronic supplementary material

MIMARKS survey

## Electronic supplementary material

**Supplementary information** accompanies the paper on the *npj Biofilms and Microbiomes* website (10.1038/s41522-018-0059-0).
